# Pancreatic Stellate Cells: A Rising Translational Physiology Star as a Potential Stem Cell Type for Beta Cell Neogenesis

**DOI:** 10.3389/fphys.2019.00218

**Published:** 2019-03-12

**Authors:** Yunting Zhou, Bo Sun, Wei Li, Junming Zhou, Feng Gao, Xiaohang Wang, Min Cai, Zilin Sun

**Affiliations:** ^1^Department of Endocrinology, Zhongda Hospital, Institute of Diabetes, School of Medicine, Southeast University, Nanjing, China; ^2^State Key Laboratory of Bioelectronics, School of Biological Science and Medical Engineering, Southeast University, Nanjing, China; ^3^Department of Outpatient, Army Engineering University, Jingling Hospital, Nanjing University, Nanjing, China; ^4^Graduate Innovation Platform of Southeast University, Nanjing, China

**Keywords:** pancreatic stellate cell, quiescent, activation, stem/progenitor cell, physiological functions, β-cells neogenesis

## Abstract

The progressive decline and eventual loss of islet β-cell function underlies the pathophysiological mechanism of the development of both type 1 and type 2 diabetes mellitus. The recovery of functional β-cells is an important strategy for the prevention and treatment of diabetes. Based on similarities in developmental biology and anatomy, *in vivo* induction of differentiation of other types of pancreatic cells into β-cells is a promising avenue for future diabetes treatment. Pancreatic stellate cells (PSCs), which have attracted intense research interest due to their effects on tissue fibrosis over the last decade, express multiple stem cell markers and can differentiate into various cell types. In particular, PSCs can successfully differentiate into insulin- secreting cells *in vitro* and can contribute to tissue regeneration. In this article, we will brings together the main concepts of the translational physiology potential of PSCs that have emerged from work in the field and discuss possible ways to develop the future renewable source for clinical treatment of pancreatic diseases.

## Introduction

Diabetes mellitus is one type of the most common chronic diseases over world, with an immense impact on public health. The number of individuals affected with this metabolic disorder is almost increased to 592 million by 2035 (Ogurtsova et al., 2017). Diabetes mellitus is caused by substantial deficits in functional β-cells in both type 1 and type 2 diabetes. Supplementation of deficient β-cells through stem cells transplantation provides ideal therapeutic effect, but this option is limited by donor sources and graft survival (Wang et al., 2015). Therefore, stem cell derived from adult pancreas that can be prompted to differentiate into insulin-secreting cells, would provide a self-healing source for β-cells neogenesis.

Adult tissue-specific stem cells are categorized as a cell type which has abilities to self-renewal and can differentiate into specialized functional cells. Based on their developmental potential, pancreatic stem cells, owing to their close relationship in term of developmental biology and anatomy advantages compared with stem cells derived from other tissue sources, have attracted a great deal of research over the last decade (Bouwens et al., 2013; Gargett et al., 2016). However, the exact cell type(s) that function as pancreatic stem cells remain unclear.

Pancreatic stellate cells (PSCs) are a multifunctional cell type found in endocrine and exocrine pancreatic tissue and comprising about 7% of parenchymal cells in the pancreas (Apte et al., 2012). PSCs can be activated into myofibroblast-like phenotype along with expression of the activation marker protein α-smooth muscle actin (α-SMA) and a reduction in the number of retinoid-containing fat droplets, and they play a key pathological role in islet fibrosis, which contributes to the progression of β-cell dysfunction (Phillips, 2012). Recently, PSCs were proposed as a potential stem cell type for β-cell neogenesis (Jiang and Morahan, 2014). PSCs express multiple stem cell markers, are multipotent and can successfully differentiated into insulin-producing cells (Mato et al., 2009; Kordes et al., 2012; Zha et al., 2016; Pang et al., 2017). Thus, to summarize the physiological role of PSCs would not only to bring an improved understanding of the biological function of PSCs in pancreas homeostasis, but would likely yield new therapeutic strategies for β-cell regeneration.

## Naming and Classification of PSCs

Pancreatic stellate cells were first identified in the mouse pancreatic duct in 1982 as a cell type enriched in lipid droplets and appearing vitamin A (VA) -specific blue fluorescence (Watari et al., 1982). In 1990, These cells were identified in healthy sections from human and rat pancreas and named pancreatic stellate cells (Ikejiri, 1990). Later in 1998, two groundbreaking advancements were reported by Apte et al. (1998) and Bachem et al. (1998) groups; they developed a method to isolate, culture, and determine the characteristic expression of PSCs, which was a useful *in vitro* tool to study the biological characteristics of PSCs in their physiological state.

The existence of PSCs in islets was debated until 2016, when our group (Zha et al., 2014; Zha et al., 2016) isolated, identified, and named the fibrogenic cells obtained from mouse, rat, and human islets using collagenase digestion, islet stellate cells (ISCs). Furthermore, we compared the biological characteristics of ISCs with typical PSCs and found that ISCs had fewer lipid droplets than PSCs, appeared to be more easily activated by stimulators, and demonstrated reduced proliferation and migration abilities compared with PSCs (Wang et al., 2018). Using single-cell transcriptome technology, recent studies further confirmed that stellate cells are present in islets (Li J. et al., 2016; Lawlor et al., 2017). These results show that ISCs should be a sub-type of PSCs and appeared to be capable of exert direct effects on islet.

**FIGURE 1:**
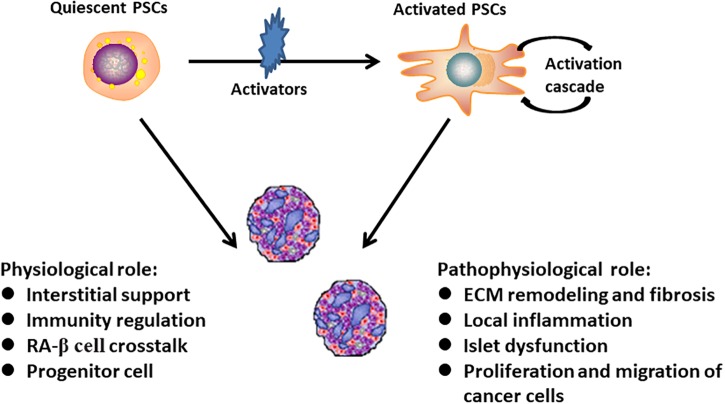
The above summarizes physiological and pathophysiological functions of different phenotypes pancreatic stellate cells. Quiescent PSCs undergo morphologic and functional changes to become activated myofibroblast-like cells. Studies have identified a variety of physiological and pathophysiological functions of PSCs in pancreas homeostasis (Masamune et al., 2008; Means, 2013; O’Byrne and Blaner, 2013; Zang et al., 2015).

Pancreatic stellate cells can be divided into two biological phenotypes. In physiological conditions, PSCs are rich in intracellular lipid droplets and positive for glial fibrillary acidic protein (GFAP) and desmin expression. These are termed quiescent PSCs. When they are activated from the resting state to myofibroblast-like cells with a concurrent disappearance of lipid droplets, they are called activated PSCs. Activated PSCs specifically express α-SMA and secreted of collagen I, collagen III, fibronectin, and other ECM components to promote the formation of pancreatic fibrosis. The presence of lipid droplets, simultaneous expression with GFAP, nestin, desmin, and vimentin is used to define the quiescent phenotype of PSCs (Nielsen et al., 2017). The detailed mechanisms about the PSCs activation and disappearance of lipid droplets have not yet well understood. In addition to a large number of cytokines, other known activators include alcohol and its metabolites, endotoxin, oxidative stress, hyperglycemia, and some factors pertinent to pancreatic injury (Bynigeri et al., 2017). The physiological and pathophysiological functions of different phenotypes PSCs were shown in Figure 1.

## Physiological Function of PSCs

Much attention has been paid to exploring the behavior of activated PSCs as a negative regulator cell type for pancreatic diseases through the production of multiple inflammatory cytokines, enhanced self-proliferation, and fibrogenesis (Bynigeri et al., 2017). However, quiescent PSCs, which proliferate rarely and express few cell-specific markers, appear stagnant. Currently very little knowledge is shown about their biological significance for tissue homeostasis.

Current opinion holds that quiescent PSCs function as intermediary cells that contribute to the parenchymal function and cell structure through maintenance of the normal basement membrane (Means, 2013). These cells often show supportive effects such as supplying blood flow and providing scaffolding for epithelial integrity (Riopel et al., 2013; Sekiguchi and Yamada, 2018). Pancreas is completely different from other organs such as the intestines, which are responsible for barrier functions and nutrient absorption. Pancreas is short of stromal layer whose vasculature travels spreads along between major ducts and acini where PSCs are located. In addition, PSCs can regulate ECM turnover by regulating synthesis via matrix degrading enzymes (Riopel et al., 2013). Quiescent PSCs also partially maintain ECM components through secretion of metalloproteinases (MMP), such as MMP-2, MMP-9, and MMP-13, as well as their inhibitors (Phillips et al., 2003). These results strongly support that the effect of PSCs in the production of the acinar basement membrane but leave the question of how much effect of quiescent PSCs has in basement membrane maintenance under homeostatic conditions.

The description above is unlikely to capture the all aspect of physiological functions of quiescent PSCs. As rat PSCs were shown to express toll-like receptors (TLR), one might suppose that stellate cells play a role in innate immunity by phagocytosis of exo- and endogenous antigens (Masamune et al., 2008). Shimizu et al. (2005) found that PSCs act as resident phagocytic cells, and that CD36 promotes peroxisome proliferator-activated receptor γ transactivation. Hepatic stellate cells (HSCs), which have many biological features in common with PSCs, expressed the MHC class II proteins required for interaction with T cells (Yin et al., 2013; Weiskirchen and Tacke, 2014). Chen et al. (2006) reported co-transplanted HSCs effectively protected isletallografts from rejection and formed as a multi-layered capsule that reduced allogeneic immunocyte infiltration by enhancing apoptosis in the islet transplantation model. These findings suggest that PSCs and phagocytic immune cells have several functions in common.

Quiescent PSCs are rich in droplets containing retinoid, predominantly as retinyl palmitate cytosolic droplets. Similarly, HSCs are responsible for up to 80% of the retinoid storage in the adult normal liver, and they can respond to VA metabolic needs in target tissue (Blaner et al., 1985; Hendriks et al., 1985). The exact function of retinol in PSCs has not been fully explored. Evidence has shown that VA and its derivatives play an important role in tissue homeostasis and the pathophysiology of pancreatic diseases, influencing cellular immunity and differentiation as well as cell apoptosis (Ross, 2012; O’Byrne and Blaner, 2013; Tejon et al., 2015). During the early days of development of the fetal pancreas, VA deficiency reduced the β-cell mass, which is attributed to a decrease in β-cell neogenesis, leading to impaired glucose tolerance in late adulthood (Matthews et al., 2004; Duester, 2008; Rhinn and Dolle, 2012). In the adult pancreas, retinoic acid (RA) is required for maintaining normal islet structure, endocrine cell mass, and endocrine function in non-diabetic mice (Kane et al., 2010; Raghow, 2012; Iqbal and Naseem, 2015). Dietary VA deprivation resulted in α-cell programmed cell death and islet remodeling, preventing the growth and proper functioning of pancreatic islets (Trasino et al., 2015). Retinoid can promote maintenance of the quiescent phenotype of PSC by inhibiting the activation of α-SMA and decreasing the expression of collagen synthesis (McCarroll et al., 2006; Chronopoulos et al., 2016; Sarper et al., 2016). All-trans RA (ATRA) has been proposed to restore and maintain the quiescent state of PSCs, suppressing their capacity to assist pancreatic exocrine and endocrine cells (Sarper et al., 2016).

**FIGURE 2:**
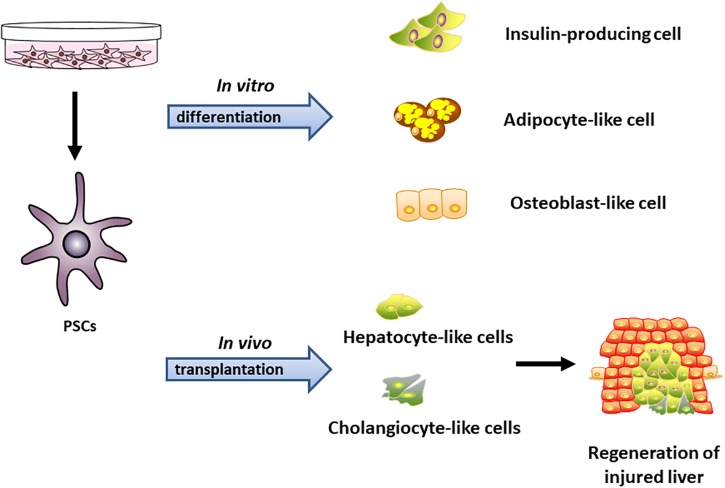
The multipotential differentiation abilities of PSCs *in vitro* and *in vivo*. PSCs can be induced to differentiate toward insulin-producing cell, adipocyte-like cell, and osteocytes-like cell *in vitro.* Transplanted PSCs were able to repopulate large areas of the host liver after partial hepatectomy in rats through differentiation into hepatocytes and cholangiocytes *in vivo* (Mato et al., 2009; Kordes et al., 2012; Zha et al., 2016).

The current evidence about the relationship between PSCs and islets has shown that PSCs not only promote islet fibrosis, but also contribute to endocrine functions in pancreatic diseases such as glucose intolerance, as well as in islet survival (Zha et al., 2014; Zang et al., 2015; Lee et al., 2017). Zang et al. (2015) found activated PSCs affected islet insulin release in short period researches in co-culture system, as measured by the secretion percentage of the cell insulin content, and increased islet cell death during prolonged culture periods. Mechanistic analyses have suggested that the production of local cytokines from macrophages in islet is the cause of this (Donath et al., 2009; Xue et al., 2015; Zang et al., 2015). We believed that the type of PSCs associated with islet dysfunction may be classified as ISCs, because activated ISCs were conducive to islet fibrosis and pancreatitis (Li F.F. et al., 2016). Therefore, more studies are needed to further evaluate the distribution of PSCs in the normal endocrine and exocrine tissues and in transplanted islets. The multipotential differentiation abilities of PSCs were shown in Figure 2.

## PSCs and Pancreatic Stem Cells

Numerous studies have identified that there are undifferentiated adult stem cells in very small numbers in the pancreas that can self-renew and differentiated into some or all of specialized cell types (Scharfmann, 2003; Pagliuca et al., 2014). Adult stem cells have been found in the intestinal epithelium, liver, skin, and hematopoietic tissue. However, the identity of pancreatic stem cells is largely unknown (Jiang and Morahan, 2014). There are various types of pancreatic interstitial cells that have been identified as potential adult pancreatic stem cells, such as nestin-positive cells (Feng and Wang, 2007), “side population” (SP) cells (Augstein et al., 2018) and so on. More recent studies have characterized a population of PSCs as likely to share a biological phenotype with these pancreatic stem cells.

### Co-localization

One reason why the identity of pancreatic stem cells cannot currently be determined with certainty is that the location of pancreatic stem cells is debated. Some researchers hold the view that pancreatic stem cells may be a subset of the mesenchymal cells that are activated during the re-emergence of embryonic islets during maintenance and repair of the pancreas. These cells are located in the pancreas interstitial or surrounding islets.

One potential stem cell type-nestin positive cells, derived from human fetal pancreas, could formed three-dimensional insulin-producing cell clusters *in vitro* and reversed hyperglycemia in animal models (Huang and Tang, 2003; Zhang et al., 2005). Eberhardt et al. (2006) have also isolated nestin positive stem cells from human islets. In accordance with a mesenchymal phenotype, the cells also able to adopt pancreatic endocrine, adipocytic, or osteocytic phenotype *in vitro* and a hepatic phenotype *in vivo*. Recent single-cell genetic analysis showed that isolated nestin positive cells have multi-mesodermal potential and would therefore conform to the definition of teno-lineage stem cells (Yang et al., 2014; Yin et al., 2016; Bai et al., 2018). Furthermore, 5-bromo-2′-deoxyuridine labeling and immunohistochemical staining revealed that nestin positive cells first appeared in the area surrounding the interlobular region and then were diffusely distributed and filled the pancreatic lobules, after which they migrated toward the pancreatic lobules using the interlobar vessels as channels and penetrated through the vascular endothelium into the pancreatic acinar tissues. A portion of the stem cells eventually penetrated into islet tissue (Gong et al., 2014). The other known potential stem cell type-SP cells-has been identified in non-hematopoietic tissues including the human pancreas (Lechner et al., 2002). Freshly SP cells did not contain insulin protein or RNA, but expressed the homeobox transcription factor *Pdx1* required for development of β-cell. SP cells also could be differentiated into insulin-expressing cells that could response to glucose (Challen and Little, 2006). In the adult pancreas, SP cells expand in response to β-cell injury and are a source of β-cell progenitors with potential for the treatment of diabetes (Banakh et al., 2012; Augstein et al., 2018). Recent evidence has shown that SP cells can be detected as a low fluorescence by flow cytometry. The fluorescence of SP cells was quite similar to PSCs, which elicit a transient blue-green fluorescence typical of VA under UV light exposure. Meanwhile on the flow two-dimensional analysis of the dot matrix, SP cells are distributed in a comet-like form on the side of the main group of parenchymal cells, whose location is very similar to that of PSCs.

Pancreatic stellate cells are located near the basolateral side of pancreatic acinar cells, around small pancreatic ducts and blood vessels (Apte et al., 2012). A single stellate cell usually has multiple processes which extend across the space of disse to make contact with parenchymal cells. This intimate contact between PSCs and their neighboring cell may promote the intercellular transport of soluble mediators and cytokines. In islets, ISCs endings are directly adjacent to endocrine cell, which is consistent with functional studies confirming the hormone responsiveness of stellate cells (Li F.F. et al., 2016; Zha et al., 2016). Therefore, SP cells, nestin-positive cells, and PSCs may have the same location. Considering the characteristics including nestin expression and auto-fluorescence in SP cells and nestin-positive cells, PSCs may represent a similar cell type in the adult pancreas that functions as stem/progenitor cells.

### Co-origin

Pancreatic development is a series of bifurcating lineage processes: ectoderm vs. mesoderm and endoderm, exocrine vs. endocrine and hormone-positive cell types vs. non-hormone-positive cell types. Throughout the pancreatic development, pancreatic epithelium is sheathed in layers of mesenchyme (Jennings et al., 2015). There are a variety of cell types in this mesenchyme, including smooth muscle, stroma, and endothelial cells (Cleaver and Dor, 2012). In terms of potential pancreatic stem cells, nestin-positive cells as well as SP cells appear to be likely possibilities given their shared mesenchymal phenotype, combined expression of several dry effectors, and close proximity *in situ*.

Since PSCs were first identified, their embryologic origin has been elusive. Thus far, no direct evidence has been obtained to identify the origin of PSCs. The results of transcriptomics and proteomics analyses showed that PSCs and HSCs have significant similarities (Paulo et al., 2013). A recent study provided striking evidence to support mesodermal origin of HSCs using a lineage analysis approach (Asahina et al., 2009; Asahina et al., 2011; Simsek et al., 2016). As most of the characteristic features and functions of PSCs are similar to those of HSCs, this suggests that both might have evolved from a mesodermal origin. However, a recent study showed PSCs was in a very small possession of VA and a low expression of lecithin retinol acyltransferase compared to those of HSCs. The microstructure of PSCs was entirely different from that of HSCs as observed by electron microscopy (Yamamoto et al., 2017). There is still no firm experimental evidence for the origin of PSCs. Nevertheless, at least a subpopulation of PSCs in the normal pancreas has been shown to have a mesodermal origin. These seemingly contradictory studies favor progenitor cells as important players in pancreatic regeneration based on stem cell.

During development, endocrine and exocrine lineages arise from the embryonic endodermal epithelium. However, the property of lineage switching in pancreatic stem cells was observed both in altered culture conditions *in vitro* and spontaneous processes *in vivo* (Cheng et al., 2010, 2015). We believe PSCs, which may derive from a common precursor cell with pancreatic stem cells, may share this property of lineage switching.

### Co-stem Cell Markers

Stem cell transcription factors are key factors that govern cell differentiation and maturation. Adult β-cell neogenesis is a dynamic process controlled by extrinsic signals from intrinsic transcription factors and non-insulin-producing cells. At present, pancreatic stem cell markers are not yet fully established. However, there is some consensus concerning a “universal” marker for the identification of pancreatic stem cells. Substantial evidence has shown that PSCs express multiple markers in common with pancreatic stem cells, including nestin, CK-19, and ATP-binding cassette superfamily G member 2 (ABCG2), which were the most commonly mentioned (Lardon et al., 2002; Yang et al., 2007; Mato et al., 2009).

Nestin, a type VI intermediate filament protein and essential transcriptional factor for the generation of endocrine cells, was originally detected in neural stem cells during development (Bernal and Arranz, 2018). Interestingly, nestin is also expressed in a variety of adult stem/progenitor cell populations (Calderone, 2012), meets criteria for adult stem cells, including proliferation, migration, and multipotency (Park et al., 2010), and shares many phenotypic markers with mesenchymal stem cells derived from the bone marrow (Bernal and Arranz, 2018). Nestin expression in the pancreas has been detected in stellate cells, pericytes, and endothelial cells (Lardon et al., 2002; Kawamoto et al., 2009). There is no consensus on the location of nestin-positive cells in different parts of the pancreas. It is generally believed that nestin is mainly expressed in islet, while CK-19 is expressed in the ductal epithelium. During embryonic development, nestin appears earlier than CK-19. Therefore, nestin-positive cells can be identified as primitive progenitor cells earlier than CK-19-positive cells.

CK-19, a family of keratins, is generally considered to be epithelial cell-specific markers. The expression of CK-19 first appears during the early embryonic stages and continues through the late embryonic and postpartum stages. After 12 weeks of embryonic development, CK-19 positive cells are concentrated in the ducts of the exocrine glands and in the undifferentiated ductal epithelium (Lane et al., 1983). It has been suggested that during pancreatic development, high levels of CK-19 expression are first present in duct-like cells, and these CK-19-positive cells are subsequently transformed into cells with endocrine and exocrine functions. Studies have shown that CK-19 is not expressed in β-cells, but more than 90% of pancreatic ductal epithelial cells express CK-19, and only 1–2% of cells can express CK-19 and nestin simultaneously. Recent studies have shown that a subset of CK-19-positive pancreatic stem cells can differentiate into islet-like cells with insulin production and acinar epithelial cells with exocrine function (Gao et al., 2003; Yan et al., 2005; Yang et al., 2007). Therefore, a small number of CK-19 positive cells in pancreatic tissue have the potential to differentiate into pancreatic endocrine and exocrine cells, which is consistent with the basic characteristics of pancreatic stem cells.

ABCG2, one of the human ABC transporters, is an important molecule in both innate and acquired multidrug resistance, in regulation of drug bioavailability, in prognostic prediction of solid malignancies, and in protecting cancer stem cells (Mo and Zhang, 2012). Present studies found that ABCG2 is expressed in the stem cells of various tissues such as bone marrow, skeletal muscle, and nerve, but not in mature cells (Fatima et al., 2012; Padmanabhan et al., 2012). These results suggested that ABCG2 may be a stem cell marker. Recently, the presence of ABCG2 has also been found in islets and acini. Others have found ABCG2/BCRPI-expressing cells among nestin-positive islet-derived progenitor cells of embryonic pancreatic tissue, which can differentiate into islet-like cell clusters (Fetsch et al., 2006). Mato et al. (2009) found that mitoxantrone-resistant cells expressing the ABCG2 transporter obtained from rat pancreata have a PSCs phenotype and are capable of secreting insulin after cell differentiation. These data indicate that ABCG2 can be used as a marker for pancreatic stem cells. However, the relationship between ABCG2-positive cells and cells positive for other pancreatic stem cell markers, such as nestin and CK-19 is not yet well understood, and further study is needed to determine whether ABCG2 is a marker for more primitive stem cells.

### Co-signaling Pathways

Signaling pathways which have major impact on stem cell differentiation is essential to help to integrate the signal inputs in order to initiate into specific lineage (Lin and Hankenson, 2011). During the past decade, Studies have several shared signaling pathways in PSCs and pancreatic stem cells. A variety of developmentally conserved signaling pathways are currently known as important control devices of stem cell fate (Blank et al., 2008). TGFβ, Wnt, Hhh, MAPK, and other pathways are significant during embryonic developmental stage for stem cell maintenance and equal proportionate growth rate among all lineages such as body patterning, cell fate determination, organogenesis, and so on (Guo and Wang, 2009). For example, TGFβ/Smad signaling pathway effect embryonic stem cells (ESCs) differentiation in the very early lineage decisions, which provides the origin of mesodermal and endodermal cells, represents the first step of ESCs differentiation (Sakaki-Yumoto et al., 2013; Xu et al., 2018).

For PSCs, the majority of intracellular signaling pathways that regulate physiological and pathological PSCs functions have also been detected. In addition to the common proliferation and migration signaling pathways, such as MAPK family, including ERK, JNK and p38 (Masamune et al., 2003; Uchida et al., 2013), the complex signaling networks involved in organogenesis, differentiation and maturation including Hhh, Shh, TGFβ, Smad and Wnt/β-catenin pathways was also determined in PSC (Lee et al., 2008; Xu et al., 2015). These signaling pathways also play a crucial role in various physiological functions and of PSCs. MAPK families are the initial pathways that precede proliferation and migration abilities of PSCs (Masamune et al., 2003). The TGFβ-Smad2/3 pathways were involved in preserving the activated phenotype and collagen synthesis (Lee et al., 2008). Other signaling pathways, such as Wnt/β-catenin, Hhh, and Shh pathways, were shown to influence mobility and differentiation of PSCs (Bynigeri et al., 2017).

To summarize these above experimental evidence, several signaling pathways were co-detected in both PSCs and pancreatic stem cells (Table 1). Thus, while these co-signaling pathways function in PSCs have not yet been established, the available data suggested these signaling pathways may the basis for PSCs to play stem cell function.

**Table 1:** A summarizing signaling pathways of PSCs and stem cells.

Signaling pathways	Functions	
	PSCs	Stem cells
MAPK	α-SMA expression, proliferation, migration, collagen synthesis	Proliferation, self-renew, differentiation
PI3K	Proliferation, migration, collagen synthesis	Proliferation, self-renew, differentiation
PKC	Collagen synthesis	Migration, self-renew
JAK/STAT	Proliferation	Proliferation, differentiation
Smads	Collagen synthesis	Migration, differentiation
Hhh	Migration	Differentiation
Shh	Migration	Differentiation
Wnt/β-catenin	Proliferation, collagen synthesis	Proliferation, self-renew, differentiation
TGF-β	Proliferation, collagen synthesis	Proliferation, self-renew, differentiation, ECM perturbation


### Co-multipotential Differentiation Abilities

Multipotential differentiation ability is an important measure to assess whether adult stem cells are able to generate an identical line of target cells with biological plasticity. Davani et al. (2007) used human islet-derived precursor cells to examine the multipotent differentiation characteristics. Under various differentiation conditions, these cells were separately induced into adipocytes, osteocytes, and chondrocytes.

For the multipotential differentiation abilities of PSCs, our previous study found that both PSCs and its subset of ISCs were able to differentiate *in vitro* into adipocyte- and osteoblast-like cells with Oil Red O or Alizarin Red S staining (Zha et al., 2016). Kordes et al. (2012) found that PSC-derived hepatocyte-like cells appeared typical hepatocyte functions including synthesize albumin along with stem/progenitor cell factors-vimentin. Transplanted PSCs isolated from enhanced green fluorescent protein-expressing rats reached the host liver and repopulated large areas of the injured organ through differentiation into hepatocytes and cholangiocytes along with long-lasting survival ability. These findings demonstrate that PSCs fulfill the essential characteristics of stem cells and can promote the regeneration of damaged organs through differentiation across tissue boundaries.

## Conclusion

In recent years, most research interest in PSCs has focused on their central role in pancreatitis and pancreatic cancer. It has become clear, however, that the function of PSCs is no longer limited with the regulation of pathologic fibrosis in the pancreas. Its important roles in health as immune and/or progenitor cells and as intermediary cells may be more significant. Given the accumulating evidence regarding the differentiation potential of PSCs, further work is necessary to fully elucidate the delicate balance between PSCs and β-cells, to determine when and how signals are exchanged between them, and to clarify the effects of these signals. An improved understanding of the supportive physiological role of PSCs would not only improve our understanding of their biological function in pancreas homeostasis, but would likely yield new therapeutic strategies for β-cell regeneration.

## Data Availability

All datasets generated for this study are included in the manuscript and/or the supplementary files.

## Author Contributions

YZ conceived and wrote the manuscript. JZ, XW, and MC critically collected and reviewed articles. WL and FG provided the data. BS and ZS conceived and designed the manuscript.

## Conflict of Interest Statement

The authors declare that the research was conducted in the absence of any commercial or financial relationships that could be construed as a potential conflict of interest.
